# Validation of Taiwan Performance-Based Instrumental Activities of Daily Living (TPIADL), a Performance- Based Measurement of Instrumental Activities of Daily Living for Patients with Vascular Cognitive Impairment

**DOI:** 10.1371/journal.pone.0166546

**Published:** 2016-11-16

**Authors:** Hui-Mei Chen, Hsiu-Fen Lin, Mei-Feng Huang, Chun-Wei Chang, Yi-Chun Yeh, Yi-Ching Lo, Cheng-Fang Yen, Cheng-Sheng Chen

**Affiliations:** 1 Department of Occupational Therapy, College of Health Science, Kaohsiung Medical University, Kaohsiung, Taiwan; 2 Department of Neurology, Kaohsiung Medical University Hospital, Kaohsiung, Taiwan; 3 Department of Psychiatry3, Kaohsiung Medical University Hospital, Kaohsiung, Taiwan; 4 Graduate Institute of Medicine, College of Medicine, Kaohsiung Medical University, Kaohsiung, Taiwan; 5 Department of Neurology, College of Medicine, Kaohsiung Medical University, Kaohsiung, Taiwan; 6 Department of Psychiatry, College of Medicine, Kaohsiung Medical University, Kaohsiung, Taiwan; 7 Department of Pharmacology, College of Medicine, Kaohsiung Medical University, Kaohsiung, Taiwan; 8 Graduate Institute of Natural Products, College of Pharmacy, Kaohsiung Medical University, Kaohsiung, Taiwan; Istituto Di Ricerche Farmacologiche Mario Negri, ITALY

## Abstract

**Objective:**

Patients with cerebrovascular diseases often presented both cognitive and physical impairment. Disability in everyday functioning involving cognitive impairment among patients may be hard to completely rely on informants’ reports, as their reports may be confounded with physical impairment. The aim of this study was to validate a performance-based measure of functional assessment, the Taiwan Performance-Based Instrumental Activities of Daily Living (TPIADL), for vascular cognitive impairment (VCI) by examining its psychometric properties and diagnostic accuracy.

**Methods:**

Ninety-seven patients with cerebrovascular diseases, including 30 with vascular dementia (VaD), 28 with mild cognitive impairment and 39 with no cognitive impairment, and 49 healthy control adults were recruited during study period. The TPIADL, as well as the Mini Mental State Examination (MMSE), Lawton-IADL and Barthel Index (BI), were performed. The internal consistency, convergent and criteria validity of the TPIADL were examined.

**Results:**

Cronbach’s alpha of the TPIADL test was 0.84. The TPIADL scores were significantly correlated with the Lawton IADL (r = –0.587, p <0.01). Notably, the TPIADL had a higher correlation coefficient with the cognitive domain of Lawton IADL (r = –0.663) than with physical domain of Lawton IADL (r = –0.541). The area under the relative operating characteristic curve was 0.888 (95% CI = 0.812–0.965) to differentiate VaD from other groups. The optimal cut-off point of the TPIADL for detecting VaD was 6/7, which gives a sensitivity of 73.3% and a specificity of 84.5%.

**Conclusion:**

The TPIADL is a brief and sensitive tool for the detection of IADL impairment in patients with VaD.

## Introduction

Vascular diseases are known to be associated with changes in cognition and functional performance [1–4]. Cognitive impairment caused by cerebrovascular disease has been termed “vascular cognitive impairment” (VCI) [5]. VCI can be divided into two conditions: vascular mild cognitive impairment (VMCI) and vascular dementia (VaD). Following Alzheimer’s disease (AD), VCI is considered to be the second leading cause of progressive and irreversible cognitive disorders. It has been estimated about 4% of prevalence rate of VMCI among general population [6]. In addition to cognitive impairment, VCI leads to severe functional impairment that affects patients and their families.

VaD may lead to greater functional impairment than other types of dementia [7, 8]. Individuals with VaD have a unique dysfunctional profile as compared with those with AD. VCI results in both physical and cognitive impairment, which may interact with each other in the earliest detectable stages [9, 10]. Hence, disability in everyday functioning among patients with VCI may originate not only from the cognitive impairment, as in AD, but also from the physical impairment. Therefore, it is not easy to ascertain the main origin of disability in everyday functioning.

Everyday functioning has been studied in depth using the questionnaires related to instrumental activities of daily living (IADL) [11, 12]. Mobility-related limitations have been found to be the most important and most specific predictors of IADL impairment, especially in the outdoor domains [13, 14]. In addition to cognitive impairment, IADL can be adversely affected by physical impairment [15]. However, the presence of physical impairment may be the main confounding factor when IADL assessment relies solely on reports from patients or informants [16]. Even knowledgeable informants may not always be able to distinguish a consequence of cognitive decline from a result of physical limitation. It is possible that IADL impairments reported by informants are more likely to be due to motor deficits than cognitive deficits. Therefore, development of an IADL assessment tool that minimizes this confounding effect is warranted.

Performance-based assessment of IADL relies on the individual’s performance in a specific context and could decrease the susceptibility to bias on the part of informants [17–19]. The Taiwan Performance-based IADL (TPIADL) is quick and easy to administer and has been validated for use as a screening tool for AD [20]. All aspects of the TPIADL using 5 tasks substantially associated with cognitive performance in three domains–memory, reasoning, and processing speed. All tasks require minimal demands on motor skill. For example, in the cooking item, it used the task of naming the ingredients on a food, rather than assessing one’s ability to prepare food. This characteristic results in a reduced effect of motor bias on the IADL score due to physical limitation in patients with cerebrovascular diseases. The TPIADL focuses on cognitive difficulties that are more apparent in executive functioning and processing speed. Thus, a validated TPIADL would have advantages in terms of functional assessment of patients with VCI, including a focus on the influence of cognitive impairment and reduction in the reporting bias of informants.

To our knowledge, few studies have performed validation of the use of a performance-based IADL instrument for the assessment of the non-AD form of cognitive impairment [11, 12]. The psychometric properties of the TPIADL were studied and the optimal cut-off point for the diagnosis of AD has been suggested [20]. This study aimed to investigate functional performance among patients with VCI using the TPIADL and analyze the differences in cognition impairment among the individuals with various levels of VCI.

## Methods

### Participants

This study was conducted in the neurological and psychiatric outpatient units of one university hospital. The inclusion criteria for the group with cerebrovascular diseases (the vascular group) were subjects 1) aged 55 years or older; 2) with sufficient language skills for completion of neuropsychological tests; 3) with an informant knowledgeable about the subject’s recent and past medical history; and 4) with a history of stroke. Study participants underwent conventional brain magnetic resonance imaging (MRI) examination. The exclusion criteria were: (1) comorbidity with other brain diseases, such as neurodevelopmental diseases, Alzheimer's disease (AD), Dementia with Lewy bodies (DLB), Parkinson's disease (PD), frontotemporal dementia (FTD), Huntington's disease (HD); infection in the central nervous system; epilepsy, and brain tumor; and (2) major psychiatric disorders, such as substance use disorder, schizophrenia, and bipolar disorder. Diagnosis of VCI was made based on the observation of cognitive changes by knowledgeable informants and confirmed by a neurologist or psychiatrist conducting the research. Cognitive changes might involve changes in memory of recent events, making decisions, handling personal matters, or language expression and comprehension. If cognitive changes did not interfere with basic activities of daily living, the VCI was classified as vascular mild cognitive impairment (VMCI), whereas if activities of daily living were affected, the condition was classified as vascular dementia (VaD). Subjects without cognitive impairment were classified as the vascular control (VC).

A healthy control (HC) sample consisting of adults of similar ages to the participants in the vascular group was also studied. The inclusion criteria for these subjects were as follows: (1) no history of stroke or transient ischemic attack; (2) aged 50 years or older; (3) possession of sufficient language skills to complete neuropsychological tests; and (4) achievement of a score of 24 or higher on the Mini Mental State Examination (MMSE). The exclusion criteria were similar to those for the vascular group.

The protocol was approved by the Institutional Review Board of Kaohsiung Medical University Hospital. Written informed consent was obtained from all subjects prior to interview. If a participant had a compromised capacity to consent, a legally-authorized family member provided written consent on behalf of the participant.

### Taiwan Performance-based IADL (TPIADL)

The TPIADL [20] was designed to assess IADL ability in five domains: communication, finance, cooking, shopping and medicine use. To allow culture- and lifestyle-specific measurement, actual and simulated everyday objects were employed as stimulus materials. Moreover, personal and environmental contexts (e.g., education level and lifestyle materials) were considered in order to prevent compromise of reliability. To develop more easily comprehensible stimuli materials, pictorial representations of a telephone directory and medication directions were used in the communication and medicine use tasks. In addition, the instrument assessed cognitive IADL ability; few motor skills were needed during the assessment, which prevented bias owing to physical impairment in the patients with VCI. Performance-based tasks were time-limited to 30 seconds, and the scoring reflected the level of efficiency in terms of the cognitive processing speed. The score for each task ranged from 1 to 3: 1 –completed without error and within 30 seconds; 2 –erroneous response within the time limit but corrected after a verbal cue; and 3 –not completed with errors within the time limit and unable to correct even following a verbal cue. The total score ranged from 5 to 15, with a higher score indicating a poorer performance in IADL.

### Other measures

The MMSE was used for assessment of cognitive function in the present study, including aspects of orientation, registration, attention, calculation, language, and recall [21]. The total score ranged from 0 to 30, with a lower score indicating poorer cognitive function. Informants reported the participants’ abilities in IADL using the Lawton-Brody IADL Scale (Lawton IADL) [22]. The Lawton IADL is a questionnaire that assesses 8 IADL functions: using the telephone, using transport, managing money, shopping, taking drugs, cooking food, housekeeping and doing laundry. The summary score ranges from 0 to 24, with a lower score indicating a poorer IADL actual performance. In the Lawton IADL, the items of using the telephone, taking drugs, and managing money could be conceptualized as cognitive IADLs (c-IADLs), while shopping, using transport, housekeeping, doing laundry, and cooking food were conceptualized as physical IADLs (p-IADLs)[23]. The Barthel Index (BI) in an informant report [24] was used for measuring basic ADL actual performance in this study. The BI is a questionnaire that consists of ten items: feeding, bathing, grooming, dressing, bowels, bladder, toilet use, transfer, mobility, and stairs. The summary score ranges from 0 to 100, with lower scores indicating a poorer basic ADL performance [25].

### Statistical analyses

Participants’ demographic data were described using means and standard deviations for continuous data, while percentages were used to report categorical variables. Chi-Square test was used to determine the group differences for gender as well as for infarction sites. Analysis of variance (ANOVA) was used to examine differences in age and education among the 4 groups. Group comparison in MMSE, BI, IADL p-IADL, c-IADL and TPIADL scores were tested with analysis of covariance (ANCOVA) controlling for age and education. Post hoc pair-wise comparisons were performed using the Tukey–Kramer tests. Since the data distribution of TPIADL, cog-IADL, IADL, phy-IADL and MMSE scores were skewed, non-parametric Spearman correlation tests were used for correlation analyses. The statistical analyses were performed using SPSS 18.0 statistical software (SPSS Inc., Chicago, IL, USA). The statistical significance level was set at p <0.05. To establish concurrent validity, correlations of the TPIADL with other measurements were analyzed by the Pearson’s correlation for entire sample. The criteria validity of the TPIADL against the diagnosis of VCI or VaD was determined via the receiver operating characteristic curve (ROC) and the area under curve (AUC); AUC ≥0.7 indicted acceptable discrimination [26]. The optimal cut-off point was estimated according to Youden’s index [27], and was valid with respect to sensitivity, specificity, positive predictive value (PPV), and negative predictive value (NPV).

## Results

A total of 97 participants of differing cognitive status were enrolled into the vascular group during a one-year study period (39 with no cognitive impairment, 28 with mild cognitive impairment, and 30 with dementia) along with 49 healthy controls.

The socio-demographic and clinical data of the participants, including MMSE, BI, Lawton IADL, p-IADL, c-IADL and TPIADL scores, are presented in Table 1. In the entire sample, the mean age was 68.7 (standard deviation [SD]: 9.2), and 55.2% of the participants were female. The duration of education ranged from 0 to 18 years (mean = 8.7 ± 4.8), and 64 (43%) participants had received fewer than 6 years of formal education. All groups had a similar gender balance. The VMCI and VaD groups were significantly older than the HC and VC groups. The HC and VC groups had significantly longer education durations than other two groups. All groups differed in terms of MMSE score, the HC and VC groups having the highest scores and the VaD group having the lowest score, which implied that the HC and VC groups had similar levels of cognition. The VaD group with the worst functional measurements on the BI, Lawton IADL, p-IADL, c-IADL and TPIADL compared with VC and VMCI. The VMCI group had worse function than the VC group according to their c-IADL and Lawton IADL performance, whereas no significant differences in BI, p-IADL and TPIADL performance. No significant difference in the performances on the BI, Lawton IADL, p-IADL, c-IADL and TPIADL between the HC and VC groups.

**Table 1:** Demographic and Clinical Data of the Study Participants.

HC group (n = 49)		Vascular group (n = 97)			p value	Post hoc#
		VC (n = 39)	VMCI (n = 28)	VaD (n = 30)		
Sex (female), n (%)	28 (57%)	18 (46%)	17 (61%)	17 (57%)	0.632	4,3>2,1
Age (years), mean (SD)	65.29 (7.50)	66.56 (7.65)	71.21 (7.33)	74.17 (11.88)	<0.001	
Education (years)	11.43 (3.20)	9.64 (4.32)	5.54 (5.0)	6.03 (4.47)	<0.001	4,3<1,2
Infarction site, n (%)						
Large vessel		8 (21%)	2 (7%)	6 (20%)		
Small vessel		12 (31%)	5 (18%)	4 (13%)		
Brainstem/cerebellum		4 (10%)	4 (14%)	3 (10%)		
White matter lesion		15 (38%)	17 (61%)	17 (57%)		
MMSE, mean (SD)	27.65 (2.15)	27.85 (1.9)	24.43 (2.92)	16.8 (6.14)	<0.001	2,1>3>4
BI, mean (SD)	99.90 (0.71)	95.77 (4.94)	92.32 (6.45)	71.83 (28.57)	<0.001	1,2,3>4
IADL, mean (SD)	23.79 (0.61)	23.08 (1.90)	20.54 (4.56)	11.17 (7.41)	<0.001	1,2>3>4
p-IADL	15.82 (0.60)	15.18 (1.64)	13.71 (3.47)	7.40 (5.32)	<0.001	1,2,3>4
c-IADL	7.98 (0.14)	7.90 (0.38)	6.82 (1.49)	3.77 (2.53)	<0.001	1,2>3>4
TPIADL, mean (SD)	5.19 (0.66)	5.44 (0.75)	6.29 (1.15)	9.43 (3.28)	<0.001	1,2,3<4

HC, healthy control; VC, vascular control; VMCI, vascular mild cognitive impairment; VaD, vascular dementia. BI: Barthel Index; c-IADL: cognitive IADL; IADL: Lawton-Brody IADL Scale; MMSE: Mini-Mental State Examination; p-IADL: physical IADL; SD: standard deviation; TPIADL: Taiwan Performance-based IADL. # 1 = HC, 2 = VC, 3 = VMCI, 4 = VaD

**Fig 1:**
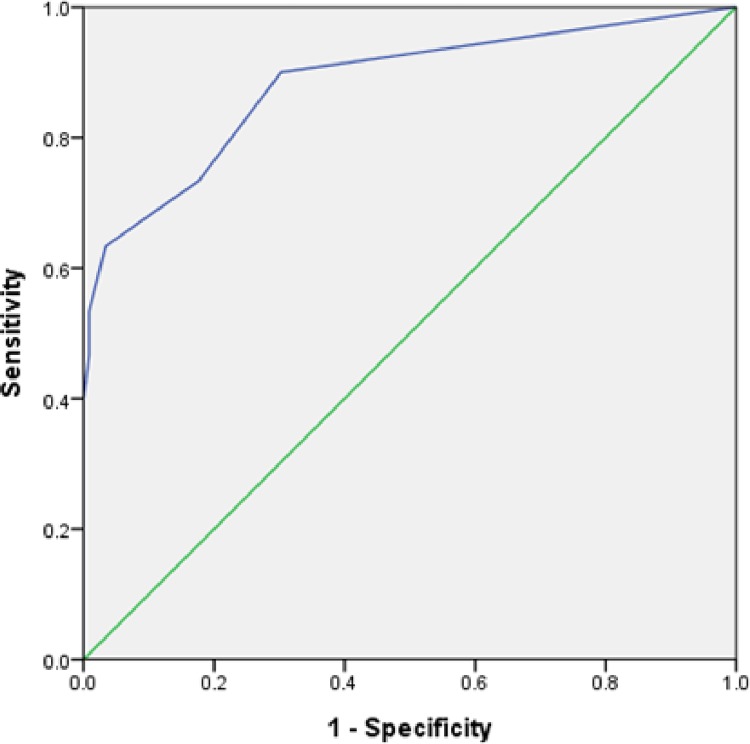
Relative Operating Characteristic (ROC) Curve for discriminating between VaD and non-VaD.

Among whole sample, the internal consistency of the TPIADL was represented by a Cronbach alpha of 0.84. The correlations between the TPIADL and the MMSE, BI, Lawton IADL, p-IADL and c-IADL scores are shown in Table 2. The total score of the TPIADL was significantly correlated with all functional status measurements. Specifically, the correlation coefficients were higher with the MMSE (r = –0.650, p <0.01) and c-IADL (r = –0.663, p <0.01), whereas the correlation coefficients were less strongly associated with the BI (r = –0.527, p <0.01), p-IADL (r = –0.541, p <0.01), and Lawton IADL scales (r = –0.587, p <0.01) (Table 2). The AUC was 0.888 (95% CI = 0.812~0.965, p <0.01) for differentiation of the VaD group from non-VaD, which indicated an excellent criteria validity for the TPIADL in differentiating those with vascular dementia from others (Fig 1). The optimal cut-off point for the TPIADL was 6/7, which resulted in validity indices as follows: sensitivity = 73.3%, specificity = 84.5%, PPV = 55%, and NPV = 93%. A similar analysis was conducted to differentiate the VMCI+VaD group from the healthy+vascular control group. The area under the ROC curve was 0.633 (95% CI = 0.529~0.737, p = 0.03), which indicated that the TPIADL was not successful in differentiating subjects with VCI from others.

**Table 2:** Correlation Coefficients between the TPIADL and Other Functional Assessments.

	MMSE	BI	p-IADL	IADL	c-IADL	TPIADL
MMSE	1.000	-	-	-	-	-
BI	0.385	1.000	-	-	-	-
p-IADL	0.531	0.467	1.000	-	-	-
IADL	0.561	0.502	0.972	1.000	-	-
c-IADL	0.623	0.580	0.785	0.864	1.000	-
TPIADL	–0.650	–0.527	–0.541	–0.587	–0.663	1.000

BI: Barthel Index; c-IADL: cognitive IADL; IADL: Lawton-Brody IADL Scale; MMSE: Mini-Mental State Examination; p-IADL: physical IADL; TPIADL: Taiwan Performance-based IADL.

## Discussion

The results of this study showed that the TPIADL, as an instrument for the evaluation of activities of daily function, had a good internal consistency. The TPIADL presented a good convergent validity with the MMSE, and had a good validity for the differentiation of patients with VaD from others. The optimal cut-off point for VaD was 6/7, with fair to good criteria validity indices, including sensitivity, specificity, PPV, and NPV. Therefore, the TPIADL can be considered a brief and effective VaD screening tool for use in Mandarin Chinese-speaking society with a generally low level of education.

Similar to our previous study of patients with AD [20], the TPIADL had a good internal consistency for use in VCI patients among the elderly population, suggesting that it is a reliable scale for the evaluation of functional impairment in these patients. Measuring IADL can be helpful in diagnosing early dementia [28]. The present results also showed that the TPIADL had a better convergent validity with the Lawton-IADL, which is the most popular and widely-applied tool for assessing IADL function, than with the BI, which indicated that the TPIADL could be used to assess IADL function in patients with VCI. Therefore, the TPIADL may be useful for early detection or for assessment in the early stages of VCI. Moreover, greater correlations were observed between the TPIADL and cognitive IADL scores than between the TPIADL and physical IADL scores. The cognitive IADL scale is more sensitive for the discrimination of dementia from non-dementia cases than the physical IADL scale [23]. This finding indicated that the TPIADL can be used as a cognitively-oriented measure of IADL function, and reinforces the TPIADL demonstrating the role of greater cognitive resources in adaptive IADL functioning. These characteristics suggest that the TPIADL has the specific advantage of reducing possible informant bias in assessing physical impairment in patients with VCI, which may distort informant-based reports. Families often struggle to differentiate the role of cognitive functioning in IADL ability, instead attributing IADL difficulties to motor deficits or other physical health problems. Therefore, the TPIADL may also be suitable for quantification of IADL performance in VCI patients with significant physical functional impairment.

There is a growing belief that performance-based measures may be more sensitive for the discrimination of cognitive impairment and normal cognitive function [29], especially in patients without proxies to provide IADL information. The results of this study revealed a good diagnostic accuracy in the discrimination of elders without VaD (normal/VC/VMCI) from those with VaD, for which the sensitivity and specificity values were 73% and 85%, respectively (AUC = 0.89). However, the results did not indicate successful differentiation between elderly adults without VCI (normal/VC) and those with VCI (VMCI/VaD), and suggested that the effectiveness of the TPIADL in terms of differentiating between VCI and non-VCI was not as strong as its effectiveness in discriminating between VaD and non-VaD in our participants. These results may also indicate that the items of the TPIADL are not sensitive enough to detect subtle change from normal to mild cognitive decline in VCI patients.

VCI poses a huge economic and social burden in developed countries [30]. It is extremely important to identify Chinese individuals with high prevalence rates of VaD than other developing countries [31], and therefore the development of improved clinical methods for early detection of VCI is critical. Although the criteria validity index did not support its use to differentiate cognitively healthy subjects from those with VCI, this brief TPIADL test could be used for initial classification of VCI patients in order to determine their need for further and comprehensive everyday function assessment, particularly on occasions at which an informant is not available.

There were some limitations of our present study. First, not all VCI patients have a documented history of stroke. Therefore, generalization of these results to VCI patients with silent strokes should be performed with caution. Second, due to the cultural variability of Chinese-speaking societies, some items that were valid in our study participants for the differentiation of individuals in different diagnostic groups may not apply to other Chinese populations. In view of the confounding effects of culture on test performance, cross-culture validation in other populations is needed. Third, not all reliability tests were conducted, such as the test-retest reliability, as this study was of a cross-sectional design.

In conclusion, the TPIADL is a brief and improved tool for the detection of VaD in older adults among Mandarin Chinese-speaking societies, particularly those with a generally low level of education. The TPIADL is suggested to be applied as screening tool for VaD and an instrument to measure IADL function in patients with a history of stroke. The TPIADL is useful even in situations in which a knowledgeable informant is not available.
